# Midaortic syndrome with IgA nephropathy in a child: a case report

**DOI:** 10.3389/fmed.2025.1567332

**Published:** 2025-09-08

**Authors:** Anna Shen, Li Shen, Wenyan Li, Huaiqing Cai, Weihua Zheng, Wenyan Huang, Rufang Zhang, Yulin Kang

**Affiliations:** ^1^Department of Nephrology and Rheumatology, Shanghai Children’s Hospital, School of Medicine, Shanghai Jiao Tong University, Shanghai, China; ^2^Department of Cardiothoracic Surgery, Shanghai Children’s Hospital, School of Medicine, Shanghai Jiao Tong University, Shanghai, China

**Keywords:** IgA nephropathy, midaortic syndrome, glomerulonephritis, vascular malformation, proteinuria

## Abstract

**Background:**

IgA nephropathy (IgAN) is one of the most common glomerulonephritis characterized by deposition of IgA immune complex in the mesangial region and mesangial proliferation in children. It could progress to end stage renal disease. The underlying mechanism of IgAN is not fully understood. It has been known that IgAN could be secondary to autoimmune disorders, respiratory tract diseases, neoplasia, infection, gastrointestinal and liver diseases. Midaortic syndrome (MAS) is characterized by narrowing or occlusion of the distal thoracoabdominal aorta and the openings of its major branches, which may lead to organ damage like heart failure, renal dysfunction and even death. It may be congenital (present at birth) or acquired later in life. So far, no cases of IgAN concomitant with MAS have been reported.

**Case presentation:**

A 12-year-old boy was diagnosed with biopsy-proven IgAN, which was characterized by heavy proteinuria and hematuria. Additionally, MAS was found by performing abdominal computed tomography angiography (CTA) due to blood pressure difference between upper arm and thigh. Methylprednisolone and cyclophosphamide were used for treating IgAN. MAS was corrected with GORE-TEX Vascular Grafts (expanded polytetrafluoroethylene, ePTFE). Remission was achieved eventually. To the best of our knowledge, we are the first to report a childhood case who has IgAN concomitant with congenital MAS. We also presented a case-based review regarding the association between IgAN and vascular stenosis.

**Conclusion:**

MAS is an aggravating factor and might be a new cause of secondary IgAN.

## Background

MAS is an uncommon cause of hypertension, primarily characterized by narrowing or occlusion of the distal thoracoabdominal aorta, as well as the openings of its major branches (1). Untreated MAS has been demonstrated to result in organ damages (2–4). Consequently, timely diagnosis and treatment are imperative. The etiology of MAS is diverse, including acquired factors like neurofibromatosis, Williams syndrome (5), Takayasu arteritis (TA), atherosclerosis (AS) and fibromuscular dysplasia, or congenital developmental abnormalities (developmental anomaly in the fusion and maturation of the paired embryonic dorsal aortas) (6). The development of effective therapeutic strategies is contingent upon accurate diagnosis, given the wide variation in treatment approaches according to the underlying cause (2). For instance, precise diagnostic assessment is required before revascularization procedures and blood pressure control measures can be implemented. Currently recognized indications for MAS treatment include: (1) aortic stenosis ≥ 60%; (2) organ perfusion insufficiency or dysfunction (such as renal insufficiency, cerebrovascular accident, left ventricular hypertrophy, or heart failure); (3) systolic pressure difference between the proximal and distal arteries ≥ 40 mmHg (7).

IgA nephropathy (IgAN) is a chronic glomerulonephritis characterized by mesangial IgA deposition and hypercellularity (8). IgAN was first reported by Berger and Hinglais in 1968 (9). It is a common glomerular disease worldwide but varies in its geographic distribution. The prevalence is highest in people of East Asian origin, followed by Caucasians, and is less common in individuals of sub-Saharan African origin (10). IgAN frequently manifests as episodes of hematuria and/or proteinuria, which is currently the most prevalent cause of hematuria of glomerular origin and primary glomerular diseases in China (11). Unlike conventional therapies, new treatments focus on the BAFF/APRIL pathways (telitacicept), complement (iptacopan), and Gd-IgA1 (Nefecon) (12). However, the underlying mechanism of IgAN is not fully understood.

IgAN includes primary and secondary ones. At present, it is known that the interaction among epigenetic, environmental and immune factors may lead to the occurrence and development of IgAN (13, 14). For primary IgAN, a well-recognized cause is mucosal infection, mostly due to respiratory and intestinal infections, resulting in the increased synthesis and abnormal glycosylation of IgA1 (15, 16). Additionally, it has been reported that patients with IgAN have vascular malformations, such as nutcracker syndrome (compression of the left renal vein) and Abernethy malformation (congenital extrahepatic portacaval shunt) (17–19). IgAN can also be complicated with vascular stenosis caused by TAK, which is a chronic inflammatory disease affecting the aorta and its main branches (20, 21). However, cases of congenital vascular malformation combined with IgAN remain unreported.

Herein, we presented a rare case of IgAN coexistence with congenital MAS. Remission was achieved after the GORE-TEX Vascular Graft operation and immunosuppressant treatment. To the best of our knowledge, it was the inaugural report of a pediatric case exhibiting both IgAN and congenital MAS. Meanwhile, a literature review regarding the association between IgAN and vascular stenosis is presented in this report.

## Case presentation

A 12-year-old boy was admitted to Shanghai Children’s Hospital in September 2021 due to the complaint of hematuria and heavy proteinuria. Prior to admission, the child had been presented with edema of both eyelids and lower limbs, as well as foamy and brown urine for two days. The edema aggravated and urine output decreased gradually. Foamy urine and gross hematuria were noted after an upper respiratory infection. His blood pressure was recorded at 175/125 mmHg (upper arm) and 102/69 mmHg (thigh). The urinalysis demonstrated the presence of proteinuria (4+), RBC > 100/HP, WBC 10–15/HP. His 24-h urine protein quantification was 12.88 g (the normal value should be less than 0.15 g), and urine protein to creatinine ratio was 12.62 (the normal value should be less than 0.2). The complete blood count (CBC) test and C-reactive protein (CRP) was normal. The values of serum creatinine, complement C3, C4, immunoglobulins, antineutrophil cytoplasmic antibodies (ANCA), autoantibodies such as antinuclear antibodies (ANA) and double-stranded DNA (dsDNA) were within the normal range. Following treatment involving a combination of immunosuppressants and antihypertensive drugs, the child’s blood pressure stabilized at approximately 145/105 mmHg and the amount of protein in their urine over 24 h decreased from 12.88 g to 11.71 g. Additionally, the patient’s estimated glomerular filtration rate (eGFR) increased from 67.0 ml/min/1.73 m^2^ to 134.1 ml/min/1.73 m^2^, returning to normal levels.

**FIGURE 1:**
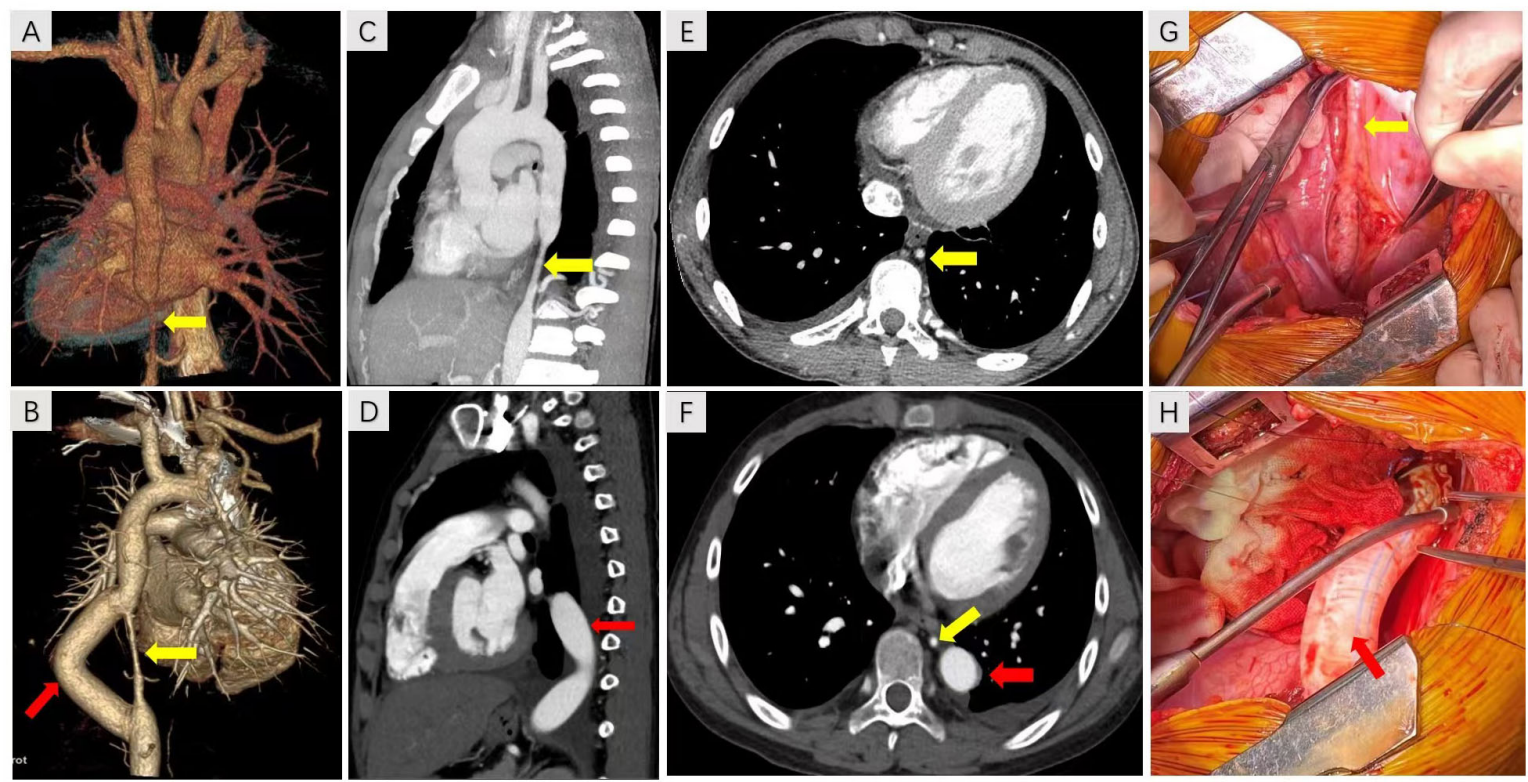
The CTA images of thorax before and after surgery. **(A,B)** The 3D reconstruction image of the stenosis (yellow arrow) and GORE-TEX Vascular Graft (red arrow). **(C–F)** The descending aortic stenosis (yellow arrow) and GORE-TEX Vascular Graft (red arrow) were visualized in the thoracic CT before and after surgery. **(G)** The stenosis of descending aorta. **(H)** A GORE-TEX Vascular Graft was used to establish a vascular bypass.

**FIGURE 2:**
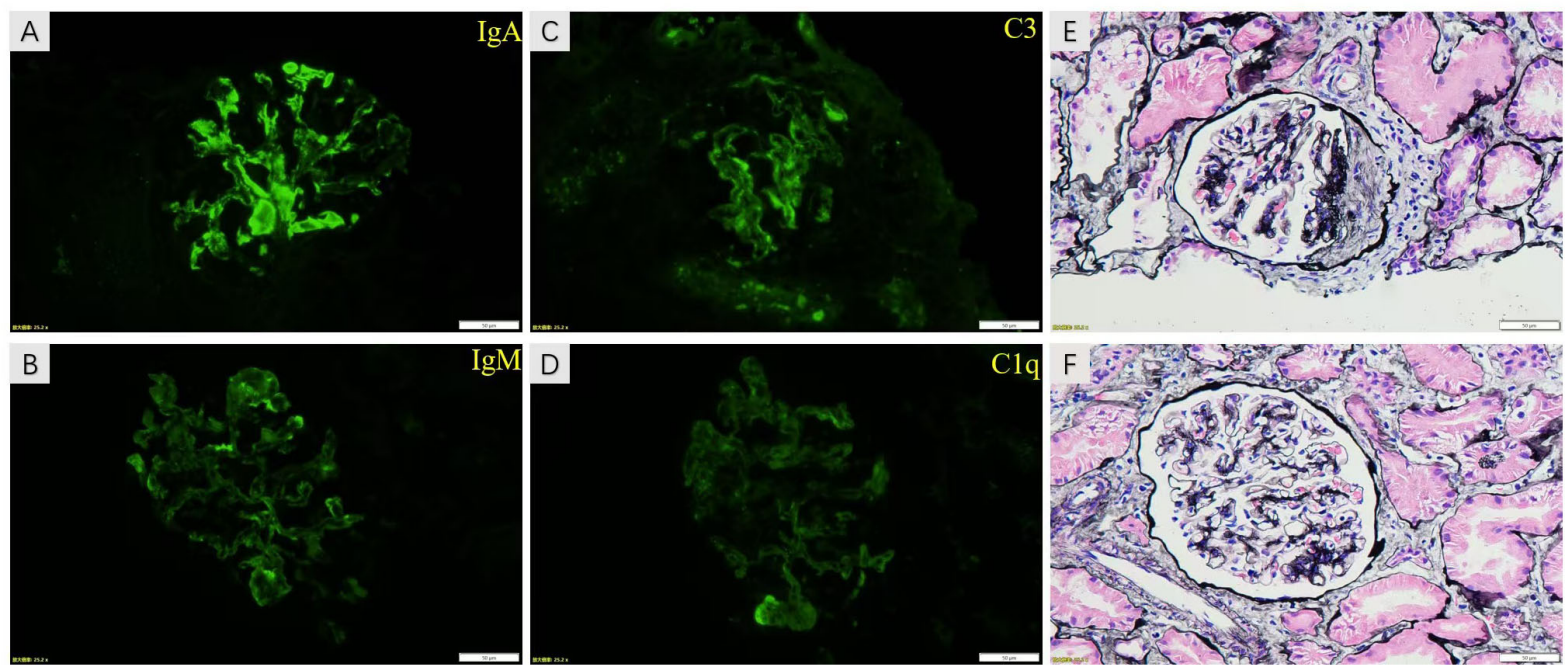
The images of renal pathology. The results of immunofluorescence staining show that IgA was strongly deposited in mesangial region **(A)**. IgM, C3 and C1q deposition were also visualized in images **(B–D)**, respectively. The proliferation of mesangial cells and matrix was shown in image **(E,F)** of PASM staining. Moreover, fibrous crescents were found in image **(F)**.

Abdominal CTA was conducted to rule out vascular malformation resulting from the discrepancy in blood pressure between the upper arm and thigh. The results demonstrated the presence of local stenosis at the lower end of the descending aorta, with a cumulative length of approximately 48 mm and a minimum lumen diameter of approximately 4.7 mm. Abnormal branch vessels were visible in both external iliac arteries. One small vessel in the left superior mesenteric artery branch exhibited proximal lumen stenosis (Figure 1), revealing the presence of MAS. The renal pathology examination results showed that among the eight glomeruli observed, one exhibited fibrous crescents, while the remaining glomeruli demonstrated focal mild to moderate mesangial cell proliferation. The results of immunofluorescence staining demonstrated that IgA was strongly deposited in mesangial region. Furthermore, IgM, C3 and C1q deposition were also observed (Figure 2). Based on these findings, a diagnosis of IgAN (M0E1S0T0C1) was made in accordance with the Oxford classification criteria (22). Subsequently, this patient received methylprednisolone, cyclophosphamide, calcium channel blocker (CCB) and angiotensin II receptor blocker (ARB) treatment. Once his condition stabilized, the patient underwent bypass graft surgery with a GORE-TEX Vascular Graft for correcting left thoracic aortic stenosis (Figure 1). GORE-TEX offers several advantages for pediatric aortic replacement surgery, including high performance, leak resistance, multiple configuration options, and excellent biocompatibility. These features make it suitable for newborns and patients with complex congenital heart disease. Because of its excellent growth potential and long-term durability, the material is ideal for artificial blood vessels and shunt surgery. Following surgery, treatment with a combination of immunosuppressants and antihypertensive drugs stabilized blood pressure at a minimum of approximately 125/65 mmHg, returning to nearly normal levels. This treatment also reduced 24-h urine protein quantification to 6.12 g, decreased the urine protein/creatinine ratio from 12.62 to 6.08 and reduced urine red blood cells (RBC) from gross hematuria to 75–100/HP. The patient’s eGFR increased from 119.5 ml/min/1.73 m^2^ to 160.0 ml/min/1.73 m^2^, both of which were within the normal range. Two years later, the child’s 24-h urine protein quantification decreased to 1.75 g during a follow-up examination at our hospital, indicating that the child achieved partial remission.

## Discussion and conclusion

IgAN is a common cause of end-stage renal disease. Secondary IgAN is always suspected if IgAN coexists with inflammatory, autoimmune disorders, infections and malignant conditions. However, the pathogenesis of secondary IgAN remains to be elucidated. Herein, we were the first to report a child with IgAN and congenital MAS. Theoretically, the ischemia and hypoxia caused by the stenosis of vessels, which is associated with renal reperfusion, may lead to kidney injury. This IgAN case presented with hypertension and a notable blood pressure discrepancy between upper and lower extremities. Eventually, the stenosis of the descending aorta was found by performing CTA.

MAS is an uncommon condition characterized by the narrowing or occlusion of the lower part of the aorta (distal thoracic or abdominal aorta, or both) along with its branches (2–4). The condition may be either acquired or congenital. MAS had a significant impact on the hemodynamics of the patients. In the congenital form, patients frequently exhibit severe hypertension during childhood, which may develop into hypertensive encephalopathy, heart failure, and stroke in adulthood (23). Furthermore, it provided an explanation for the hypertension observed in this child, despite the absence of renal dysfunction. Even though cases of renal insufficiency caused by MAS have not been reported, it still can be reasonably inferred that the stenosis of the aorta may affect the blood flow to the kidneys. Similarly, renal hypoperfusion is a prevalent complication in patients with aortic dissection (24). In the episode of aortic dissection, the blood pressure in the pseudo-vascular lumen is elevated, leading to compression of the true aortic lumen and subsequently inadequate perfusion and damage to downstream organs. Although there is no direct correlation between MAS and the dramatic reduction in renal perfusion caused by aortic dissection, prolonged stenosis inevitably leads to chronic renal ischemia. From this perspective, it suggests that MAS may lead to renal ischemia, which could be an important cause of IgAN. Moreover, ischemia and hypoxia in the kidney activate the renin-angiotensin-aldosterone system (RAAS), which may aggravate renal injury. Meanwhile, ischemia and hypoxia also initiate abnormal inflammatory responses to the release of cytokines and chemokines (25).

The current consensus on the core pathogenesis of IgAN is the “Four-Hit Hypothesis,” and MAS may promote this process through the following pathways. Firstly, the intestinal mucosa is the primary source of IgA1 (26). Ischemia has been demonstrated to result in impaired intestinal barrier function, dysbiosis, and activation of mucosal B cells, which, in turn, may lead to abnormal secretion of Gd-IgA1 (27). Secondly, the process of ischemia-reperfusion injury has been demonstrated to result in the release of intracellular antigens, including HSP70, which has been shown to disrupt immune tolerance and promote the production of IgG/IgA antibodies against Gd-IgA1 (28). Subsequently, MAS causes impairment to hepatic sinusoidal endothelial cell function, reducing the liver’s capacity to clear immune complex (IC), resulting in the retention of Gd-IgA1-IgG complexes in circulation. Finally, deposited ICs activate complement via the mannose-binding lectin (MBL) pathway and the alternative pathway (C3a, C5a), recruiting macrophage infiltration (29, 30).

It has been reported that the expression of kidney injury molecule (KIM)-1 was increased in patients with IgAN and exhibited a positive correlation with the decreased renal function. *In vitro* cell models have demonstrated that hypoxia induces the expression of KIM-1. KIM-1-expressing cells produce more chemokines/cytokines when they are cultured under hypoxic conditions (31). Another study demonstrated that the level of hypoxia inducible factor-1α (HIF-1α) can predict renal function impairment and is associated with IgAN progression (32). HIF-1α is a factor directly related to ischemia and hypoxia, which plays an important regulatory role in cell metabolism, apoptosis and inflammatory response under hypoxic conditions. The Oxford classification of IgAN has been confirmed to be closely related to the progression and prognosis of the IgAN in the majority of studies (33). Therefore, ischemia-hypoxia caused by narrowed vessels associated with renal reperfusion may be a contributing factor in the development of IgAN.

In a literature review, it has been reported that vascular stenosis caused by TAK may coexist with IgAN (34–37). TAK is a chronic inflammatory disease affecting the aorta and its main branches and one of the causes of acquired MAS. Aortic wall stenosis, occlusion and dilation may result in a range of symptoms, including pulselessness, aortic regurgitation, hypertension, and headaches. The production of inflammatory factors may be involved in the pathogenesis of IgAN caused by vascular stenosis secondary to TAK. It has been known that IL-6 plays critical roles in TAK (38). A previous study showed that IL-6 released in mucosal infections promoted the synthesis of Gd-IgA1 via modulation of key glycosyltransferases. It may also explain the increased immune-complex formation in IgAN (39). On the other hand, hemodynamic changes can also cause autoimmune dysfunction. In a recent study, pressure overload HF induced an autoimmune-like response to myocardial autoantigens in mice undergoing transverse aortic constriction (TAC) surgery, and inhibition of the autoantigen response partially ameliorated pressure overload-induced heart failure (40). The TAC model was constructed by ligating the aortic transversally and there are some similarities with our MAS case. There was a significant increase in CD4+ cells following TAC, suggesting that an antigen-specific immune response developed. A number of previous studies have found that ischemic injury-induced cell death releases cardiac antigens that elicit an autoimmune response via activated dendritic cells (DCs), again suggesting a correlation between hemodynamic alterations and autoimmunity (41, 42). Although cases of MAS leading to other organ-immune diseases have not yet been reported, this study suggests to us that constriction of the aorta may induce autoimmune reactions in the kidney.

Regarding the treatment plan for the patient, we believe correcting the vascular malformations and improving hemodynamics would benefit the patient. Prior to surgery, the patient’s blood pressure stabilized at approximately 145/105 mmHg with treatment involving a combination of immunosuppressants and antihypertensive medications. However, proteinuria control was suboptimal, with 24-h urine protein levels decreasing from 12.88 g to 11.71 g after the first round of immunosuppressive therapy. Following surgery and combined treatment with immunosuppressants and antihypertensives, the patient’s blood pressure stabilized at approximately 125/65 mmHg and the 24-h urine protein level decreased to 6.12 g, indicating that surgery to correct aortic coarctation can improve blood pressure and urine protein levels when combined with the same doses of immunosuppressants and antihypertensives. Though the antihypertensive drug formulations differed between the two rounds of immunosuppressive therapy and the doses were slightly adjusted, significant improvements in blood pressure and urine protein levels were observed before and after surgery. Additionally, prior to surgery, after one round of immunosuppressive therapy, the patient’s estimated glomerular filtration rate (eGFR) increased from 67.0 to 134.1 mL/min/1.73 m^2^. Following surgery and the same dose of immunotherapy, the patient’s eGFR increased from 119.5 to 160.0 mL/min/1.73 m^2^. Immunosuppressive therapy is undoubtedly crucial for controlling the patient’s condition, as their eGFR returned to normal after the first immunotherapy session. However, since renal function recovered after the first round of therapy, the role of descending aorta stenosis surgery in recovery could not be assessed.

In our reported case, IgAN coexisted with congenital MAS. Even though the correlation between them remains to be determined, it is plausible that the occurrence of ischemia and hypoxia due to inadequate renal perfusion, caused by vascular stenosis, may result in renal injury. Furthermore, the process of aortic constriction may give rise to an autoimmune response, thereby exacerbating and even inducing IgAN. While this case highlights a potential association between MAS and IgAN, further research is needed to determine whether MAS is a causative factor or coincidental in the pathogenesis of secondary IgAN.

## Data Availability

The raw data supporting the conclusions of this article will be made available by the authors, without undue reservation.
